# The Flesh and Milk of Tuberculous Cow

**Published:** 1882-10

**Authors:** 


THE QUESTION WHETHER THE FLESH AND MILK OF TUBERCULOUS COWS can transmit tuberculosis when eaten by man, continues to be a burning one. It is now, however, in a fair way to be definitely settled. A commission has been appointed in Germany, with Virchow at its head, to investigate the matter. A second commission is soon to be appointed by the Paris Academy of Medicine with the same object in view. In England, Drs. Simon Burdon-Sanderson and Creighton are studying the question.

In this country, a report was made not long ago by three veterinarians of this city upon the subject of tuberculosis. The conclusions reached were very novel and radical; so much so as to excite some doubts as to the capacity of the investigators.

